# SARS-CoV-2-related Guillain–Barré syndrome requires comprehensive diagnostic and therapeutic care

**DOI:** 10.25122/jml-2024-0052

**Published:** 2024-01

**Authors:** Josef Finsterer

**Affiliations:** 1 Neurology Department, Neurology & Neurophysiology Center, Vienna, Austria

**Keywords:** SARS-CoV-2 infection, polyradiculitis, Guillain–Barré syndrome, nerve conduction, demyelination

We have read with great interest the case series by Jumagaliyeva *et al*. regarding Guillain–Barré syndrome (GBS) following SARS-CoV-2 infection [1]. However, we wish to highlight several areas of concern that necessitate further discussion.

Patient 1, a 23-year-old female patient, experienced paraparesis and tingling in the lower limbs, had a demyelinating lesion in nerve conduction studies (NCSs) and showed a beneficial response to intravenous immunoglobulins (IVIGs). Patient 2, a 50-year-old female, presented with more severe symptoms, including bilateral ptosis, facial palsy, dysphonia, dysphagia, quadriplegia, and tingling, and had a demyelinating lesion on NCSs. Despite administration of IVIGs and treatment in the ICU, the patient died.

The diagnosis of GBS in Patient 1, attributed to SARS-CoV-2, overlooks the possibility of other etiological agents. Patient 1 had a history of urinary tract infection three weeks before GBS onset. Which microbial pathogen caused the urinary tract infection? The most common causes of GBS besides SARS-CoV-2 are *Campylobacter jejuni*, herpes virus, Epstein–Barr virus, cytomegalovirus, varicella-zoster virus, *Mycoplasma pneumoniae*, and Zika virus. Has the patient been tested for all these possible triggers of GBS?

One limitation of the study is that Patient 1 did not undergo cerebrospinal fluid (CSF) testing [1]. According to the Brighton criteria, diagnosing GBS requires a typical clinical presentation and NCSs and documentation of elevated CSF protein without pleocytosis and blood-brain barrier dysfunction (albuminocytological dissociation).

We also disagree with the diagnosis of Miller-Fisher syndrome (MFS) in Patient 2. MFS is typically characterized by the triad of decreased tendon reflexes, ophthalmoparesis, and ataxia [2]. Since Patient 2 only had decreased tendon reflexes, but none of the other two characteristics, the specificity of the MFS diagnosis is questionable without the supportive evidence of elevated GQ1b antibodies [2].

It was reported that respiratory failure led to death in Patient 2 [1]. Was respiratory failure due to SARS-CoV-2 pneumonia, pulmonary embolism, pulmonary hypertension, heart failure, or involvement of the respiratory muscles in GBS? Was the patient mechanically ventilated? Did she require extracorporeal membrane oxygenation? We should know whether plasma exchange or apheresis was attempted in addition to IVIGs.

To sum up, the excellent study has limitations that should be addressed before conclusions are drawn. Clarifying the weaknesses would strengthen the conclusions and improve the study. All alternative etiologies must be excluded before GBS can be attributed to SARS-CoV-2. Patients with GBS with a rapidly progressive course require immediate, aggressive, and comprehensive treatment.
